# Alpha and beta-Thalassemia mutations in Hubei area of China

**DOI:** 10.1186/s12881-019-0925-5

**Published:** 2020-01-06

**Authors:** Yaowu Zhu, Na Shen, Xiong Wang, Juan Xiao, Yanjun Lu

**Affiliations:** 1 Department of Laboratory Medicine, Tongji Hospital, Tongji Medical College, Huazhong University of Science and Technology, Wuhan, 430030 China; 2 Department of Obstetrics and Gynecology, Tongji Hospital, Tongji Medical College, Huazhong University of Science and Technology, Wuhan, 430030 China

**Keywords:** Thalassemia, Globin mutation, Prevalence Spectrum, Hubei region

## Abstract

**Background:**

Thalassemia is a group of inherited hemoglobic disorders resulting from defects

in the synthesis of one or more of the hemoglobin chains, which is one of the most prevalent inherited disorders in southern China. Only few studies reported the molecular characterization of α- and β-Thalassemia in Hubei Province in the central of China.

**Methods:**

A total of 4889 clinically suspected cases of thalassemia were analyzed by Gap-PCR, PCR-based reverse dot blot (RDB).

**Results:**

1706 (33.8%) subjects harbored thalassemia mutations, including 539 (11.0%) subjects with α-thalassemia, 1140 (23.3%) subjects with β-thalassemia mutations, and 25 (0.51%) subjects with both α- and β-thalassemia mutations. Seven genotypes of α-thalassemia mutations and 29 genotypes of β-thalassemia mutations were characterized. --^SEA^/αα (66.05%), −α^3.7^/αα (24.12%), and -α^4.2^/αα (3.71%) accounted for 93.88% of the α-thalassemia mutations. βIVS-II-654/βN, βCD41–42/βN, βCD17/βN, βCD27–28/βN, βCD71–72/βN, β − 28/βN, β − 29/βN, βCD43/βN, βE/βN, accounting for 96.40% of all β-thalassemia genotypes. Furthermore, mean corpuscular volume (MCV) and mean corpuscular Hb (MCH) were sensitive markers for both β-thalassemia and α-thalassemia with --^SEA^/αα, but not -α^3.7^/αα and -α^4.2^/αα. **Conclusions:** Our data indicated great heterogeneity and extensive spectrum of thalassemias in Hubei province of China.

## Background

Thalassemia is a group of autosomal recessive disorders with varied phenotype, which are caused by human globin gene synthesis disorders [1], including α-thalassemia and β-thalassemia [2]. No effective treatment for patients with severe thalassemia has been reported, except bone marrow transplantation, which creates an enormous burden on the family and the society. Application of thalassemia carrier screening and prenatal diagnosis to prevent the delivery of newborn with severe thalassemia is important.

In China, thalassemia is most frequent in Guangdong, Guangxi, Hainan provinces [3, 4]. The prevalence of α-thalassemia, β-thalassemia, and both α- and β-thalassemia was 8.53, 2.54, and 0.26% respectively in a cohort of 13,397 consecutive samples from Guangdong province [5]. In Guangxi province, the prevalence varied to be 24.07, 15.35, and 6.64% in 47,500 screened individuals from Baise region [6]. In Hainan province, prevalence of α-thalassemia increased to 53.45% in 8600 subjects of the Li people, however, it was only 12.16% in 9800 Han people [7]. These studies reported diverse spectrums of globin mutations in different geographical distribution regions with different ethnic populations [8]. Ethnic background and screening strategy may partially explain these differences.

**Fig. 1:**
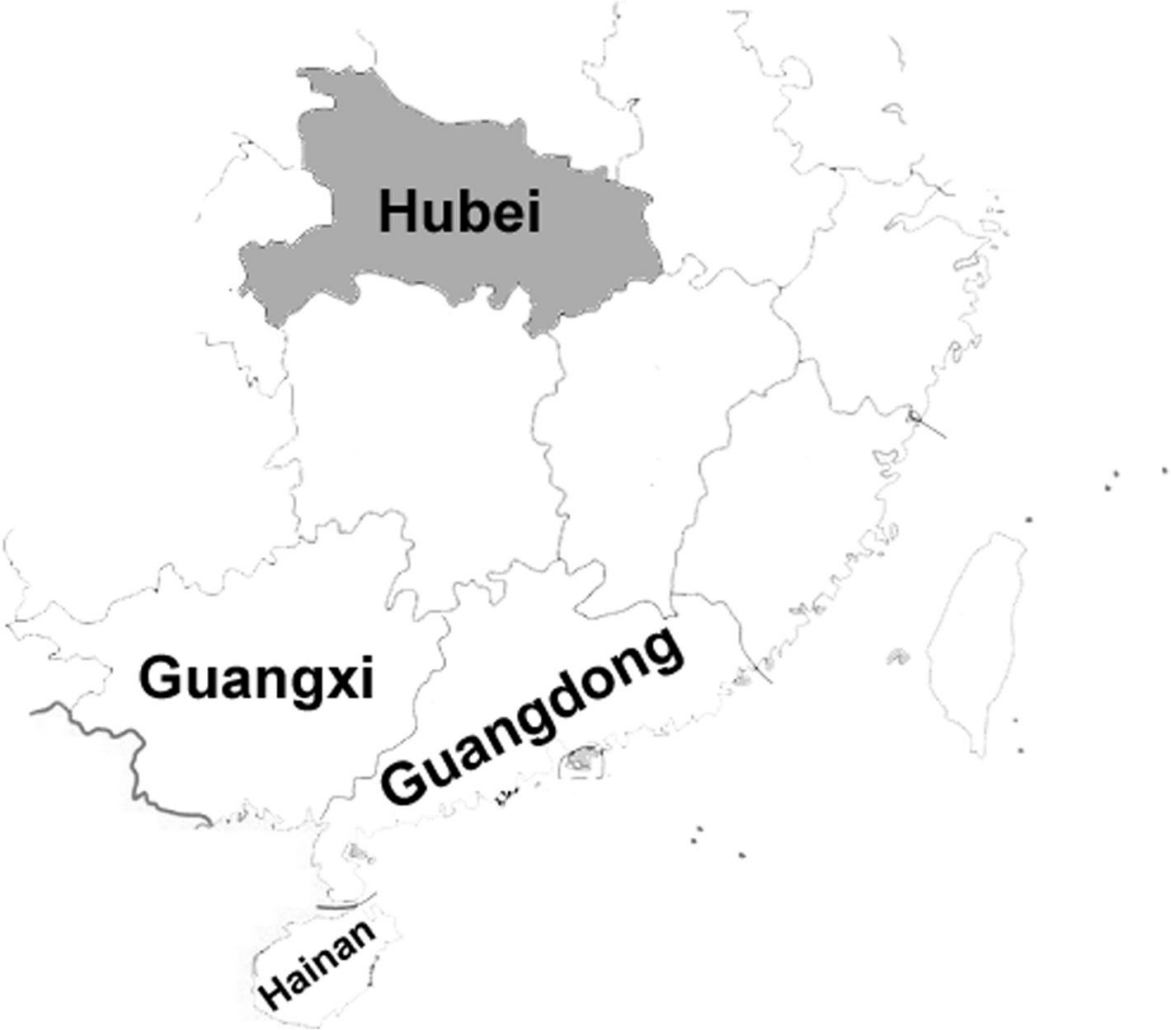
The part of map of southeastern China, which was drawn by Photoshop 6.0 software

Hubei province is located in central of China and the Han people accounts for the majority. Hubei is about 1000 km away from Guangxi and Guangdong provinces (Fig. 1). The prevalence of α-thalassemia and β-thalassemia in neonates has been investigated by Cai WQ et al., [9] and Xiong Q et al., [10] respectively in Wuhan area of Hubei province. Herein, we performed a largescale survey to reveal the α- and β-thalassemia mutations in 4889 suspected cases of thalassemia in Hubei province including both Wuhan area and around cities.

## Methods

### Human subjects

From January 2013 to October 2018, 4889 suspected cases of thalassemia (1884 males and 3005 females) had molecular testing for thalassemia from departments of Paediatrics, Hematology, and Obstetrics & Gynecolog in our hospital. The relatives of patients of thalassemia were excluded. The inclusion criteria include: (1) microcytosis and hypochromia blood routine test, or (2) positive family history. The age of the patients ranged from 1 to 88 years old. Information records of sex, age and related medical examination were available in our Electronic Medical Record Information System. This study was approved by the Medical Ethics Committee of Tongji Hospital, Tongji Medical College, Huazhong University of Science and Technology. All procedures were carried out in accordance with ethical guidelines for human subject’s research.

### Hematological analysis

Two ml peripheral venous blood samples were collected with EDTA anticoagulants, and determined with SYSMEX XS800i automatic blood cell analyzer (Kobe, Japan).

### Genetic analysis

The DNA was extracted using the DNA blood extraction kit (Tiangen Bio-Tech Co. Ltd., Beijing, China) according to the manufacturer’s instructions. Gap-polymerase chain reaction (gap-PCR) and PCR-reverse dot-blot assays were used to analyze α-thalassemia and β-thalassemia mutations with commercial kits from Yaneng Biosciences (Shenzhen, Guangdong, China) as previously described [11].

## Results

One thousand seven hundred six subjects were detected with thalassemia mutations, including 539 (11.0%) subjects of α-thalassemia mutation alone, 1140 (23.3%) subjects of β-thalassemia mutation alone and 25 (0.51%) subjects of both α- and β-thalassemia mutations.

Among the 539 cases with α-thalassemia mutations, seven types of α globin gene deletion were detected (Table 1). --^SEA^/αα was the most frequent genotype, accounting for more than half of all α-thalassemia genotypes (66.05%). -α^3.7^/αα was the second highest genotype and accounted for 24.12%. In addition, the proportion of -α^4.2^/αα was 3.71%, and the proportions of compound heterozygous -α^3.7^/−−^SEA^, −α^4.2^/−−^SEA^ genotype were 3.53, 1.67% respectively. Also a small proportion of the homozygous -α^3.7^/−α^3.7^ (0.74%) and -α^4.2^/−α^4.2^ (0.19%) were found in our study.

**Table 1:** Genotype, number of subjects and frequency of thalassemia deletions

Genotype	*n*	Frequency (%)
α-thalassemia		
--^SEA^/αα	356	66.05
-α^3.7^/αα	130	24.12
-α^4.2^/αα	20	3.71
-α^3.7^/−α^3.7^	4	0.74
-α^4.2^/−α^4.2^	1	0.19
-α^3.7^/−−^SEA^	19	3.53
-α^4.2^/−−^SEA^	9	1.67
Total α-thalassemia	539	100
β-thalassemia		
Heterozygote		
βIVS-II-654/βN	546	47.89
βCD41–42/βN	284	24.91
βCD17/βN	158	13.86
βCD27–28/βN	32	2.81
βCD71–72/βN	28	2.46
β − 28/βN	20	1.75
βCD43/βN	13	1.14
β − 29/βN	10	0.88
βE/βN	8	0.7
βCD14–15/βN	3	0.26
βIVS-I-1/βN	3	0.26
βIVS-1-5/βN	2	0.18
βcap	2	0.18
Total β-thalassemia	1109	97.28
Compound heterozygote		
βCD41–42/βIVS-II-654	5	0.44
β −28/βIVS-II-654	4	0.35
βCD17/βIVS-II-654	3	0.26
βCD41–42/βCD17	1	0.08
βCD71–72/βCD14–15	1	0.08
β-29/βIVS-II-654	1	0.08
βE /βIVS-II-654	1	0.08
βCD27–28/βIVS-II-654	1	0.08
βE /βCD27–28	1	0.08
β-28/βCD17	1	0.08
βCD71–72/βCD17	1	0.08
Total Compound heterozygote	20	1.75
Homozygote		
βIVS-II-654/βIVS-II-654	6	0.53
βCD41–42/βCD41–42	2	0.18
βCD17/βCD17	1	0.08
β − 29/β − 29	1	0.08
βCD27–28/βCD27–28	1	0.08
Total Homozygote	11	0.96
α- and β-thalassemia		
-α^3.7^/αα and βIVS-II-654/βN	7	28
-α^3.7^/αα and βCD41–42/βN	4	16
--^SEA^/αα and IVS-II-654/βN	4	16
--^SEA^/αα and βCD41–42/βN	3	12
-α^4.2^/αα and IVS-II-654/βN	2	8
-α^3.7^/αα and βCD17/βN	1	4
-α^3.7^/αα and βE/βN	1	4
--^SEA^/αα and βCD17/βN	1	4
--^SEA^/αα and βIVS-I-5/βN	1	4
-α^3.7^/−−^SEA^ and βCD41–42/βN	1	4
Total α- and β-thalassemia	25	100

Of the 1140 cases who were with β-thalassemia mutations, twenty-nine different genotypes were found, including 1109 heterozygotes, 20 compound heterozygotes and 11 homozygotes, accounting for 97.28, 1.75 and 0.96%, respectively (Table 1). βIVS-II-654/βN was the most common genotype, accounting for 47.89% of all β-thalassemia genotypes. Most of the remaining genotypes were βCD41–42/βN, βCD17/βN, βCD27–28/βN, βCD71–72/βN, β − 28/βN, β − 29/βN, βCD43/βN, βE/βN. Overall, these nine genotypes accounted for 96.40% of all β-thalassemia genotypes. The most common compound heterozygous genotype was βCD41–42/βIVS-II-654 (0.44%), and the most common homozygous mutation was βIVS-II-654/βIVS-II-654 (0.53%) in Hubei region.

In the 25 cases that had been found to carry compound α/β-thalassemia mutations, 10 types of gene mutation combinations were revealed, the specific phenotype, frequency was shown in Table 1. Among them, the three most common compound genotypes were -α^3.7^/αα and βIVS-II-654/βN, −α^3.7^/αα and βCD41–42/βN, --^SEA^ /αα and IVS-II-654/βN and accounted for 60%. In addition, one case with the compound α-thalassemia mutation -α^3.7^/−−^SEA^ and the β-thalassemia heterozygote βCD41–42/βN was detected in our study.

Then the relationships between the genotypes of α/β- globin mutation and the characteristics of thalassemia mean corpuscular volume (MCV) or mean corpuscular Hb (MCH) were observed. 407 patients (407/539) with α-thalassemia mutation and 867 patients (867/1140) with β-thalassemia mutation had been taken with the hematological analysis. Of the patients with --^SEA^ /αα, the level of MCV and MCH in most cases (97.4%) were lower than the normal reference interval (*MCV* 82–100 fL, *MCH* 27.0–34.0 pg), only seven cases had the normal MCV and MCH value (2.6%). While among the patients with -α^3.7^/αα, the proportions of the patients with the normal MCV or MCH value were 36.3, 35.2% respectively, also the similar results were seen in the patients with -α^4.2^/αα genotype (Table 2).

**Table 2:** Genotype and hematologic data of thalassemia patients

Genotype	MCV < ref	MCV nor	MCH < ref	MCH nor
α-thalassemia				
--^SEA^/αα	267	7	267	7
-α^3.7^/αα	58	33	59	32
-α^4.2^/αα	10	7	10	7
Compound heterozygote	23	0	23	0
homozygote	1	1	1	1
β-thalassemia				
βIVS-II-654/βN	383	23	390	16
βCD41–42/βN	200	7	201	6
βCD17/βN	123	5	124	4
βCD27–28/βN	26	0	26	0
βCD71–72/βN	21	1	21	1
β-28/βN	14	3	15	2
βCD43/βN	9	1	10	0
βE/βN	6	2	6	2
β-29/βN	4	2	4	2
βCD14–15/βN	3	0	3	0
Compound heterozygote	15	3	14	4
homozygote	8	2	4	6
Rare variant	3	2	3	2

## Discussion

In this study, the thalassemia data were obtained through a large sample of 4889 patients in our hospital from Hubei province, the central region of China. The common thalassemia genotype status in this region was described. The overall frequency of thalassemia mutation carriers was 33.81%, 11.0% of subjects were α-thalassemia mutation carriers, 23.3% of subjects were β-thalassemia mutation carriers, and 0.51% of subjects were both α- and β-thalassemia mutation carriers. The carrier frequency here was higher than that in previous reports in Guangdong (11.33%) [5] and Guangxi (24.51%) [12], especially the genotype distribution was different from their reports that the proportion of α-thalassemia mutation was near two fold than β-thalassemia mutation. This discrepancy was probably due to the testing strategy and in the population selected, in our study, the subjects were mainly suspected with thalassemia, most of them with microcytosis, while in some studies, healthy subjects coming for routine healthy examination [6]. Difference in ethnicity may also partially explain the differences. In Guangxi and Hainan, Baise descents or Zhuang people and Li people were included, in our study, mainly Han people were tested.

α-thalassemia is mainly caused by three types of genotypes, including Southeast Asian deletion (--^SEA^ deletion), the right deletion (−α^3.7^), the left deletion (−α^4.2^) in Chinese populations [13]. In the current study, seven α-thalassemia genotypes were detected, including --^SEA^/αα, −α^3.7^/αα, −α^4.2^/αα, −α^3.7^/−α^3.7^, −α^4.2^/−α^4.2^, −α^3.7^/−−^SEA^, −α^4.2^/−−^SEA^. The top genotypes were --^SEA^/αα, −α^3.7^/αα and -α^4.2^/αα, accounted for 66.05, 24.12 and 3.71%, composing of 93.88% of all the α-thalassemia mutation carriers. The compound heterozygous -α^3.7^/−−^SEA^, −α^4.2^/−−^SEA^ deletions and the homozygous -α^3.7^ /−α^3.7^ and -α^4.2^/−α^4.2^ deletions accounted for the remaining 6.12%. Consistent with previous studies, --^SEA^ deletion was the main α-thalassemia genotype, similar with that in the high incidence provinces such as Guangdong Province [14], Guangxi [15], and the adjacent Hunan Province [16].

β-thalassemia is mainly caused by point mutations in Chinese populations. The most common mutations include CD41–42, IVS-II-654, CD17, − 28 and − 29, accounting for more than 90.0% of all β-thalassemia mutations in Chinese population [17]. Twenty-nine β globin mutations were detected here. IVS-II-654 and CD41–42 accounted for 47.89 and 24.91%, respectively, followed by CD17, CD27–28 and CD71–72 (13.81, 2.81, and 2.46%, respectively). The distribution of mutation types was significantly different compared with previous reports. For example, CD17 was the most frequent β-thalassemia mutation with an allele frequency of 40.22% in this Baise region in Guangxi [6], where CD41–42 was the most common mutation in Guangdong [18, 19], and the first two mutations in Hainan Province were CD41–42, and IVS-II-654 [7], the top three mutations in Yunnan province were CD26, CD17 and CD41–42 [20]. The above results showed that the distribution of β-thalassemia mutations had strongly regional and racial specificity, showing different distribution characteristics in different regions and ethnicities.

It has been shown that the characteristic of the thalassemia carriers is microcytosis. The sensitivity of MCV and MCH for --^SEA^ /αα, −α^3.7^/αα, and -α^4.2^/αα was 97.4, 63.7, and 58.8% respectively. Patients with compound heterozygous mutations, the sensitivity of both MCV and MCH increased to 100%. Compared to the α-thalassemia carriers, the overall sensitivity is higher for MCV and MCH screening in β-thalassemia carriers.

Additionally, twenty-five cases with compound α/β-thalassemia mutations were detected in the current study, −α^3.7^/αα and βIVS-II-654/βN was the most compound α/β- thalassemia. Fourteen of them had been with hematological analysis, only eight cases showed mild thalassemia (data not shown). Our results partly support the view that the coinheritance of α-thalassemia could ameliorate the severity of β-thalassemia because it lowers α-globin production and reduces the damage to red cells caused by free intracellular α-globin [21].

## Conclusion

In conclusion, our data indicated great heterogeneity and extensive spectrum of thalassemias in Hubei province of China.

## Data Availability

The datasets generated and/or analysed during the current study are available in the [Pan.baidu.com] repository, [https://pan.baidu.com/s/1cVou7HaMGEQXcr-_GrfM6g. access code: qpdz].

## Abbreviations

MCV: Mean corpuscular volume
MCH: Mean corpuscular Hb
RDB: PCR-based reverse dot blot
